# Supplementary material for: Error-correcting dynamics in visual working memory
Source: Nat Commun. 2019 Jul 29;10:3366. doi: 10.1038/s41467-019-11298-3 (PMC6662698; doi:10.1038/s41467-019-11298-3)
Supplement: Supplementary file 1 — Supplementary Information [file 41467_2019_11298_MOESM1_ESM.pdf]

Supplementary information

Error-correcting dynamics in visual working memory

Panichello et al.

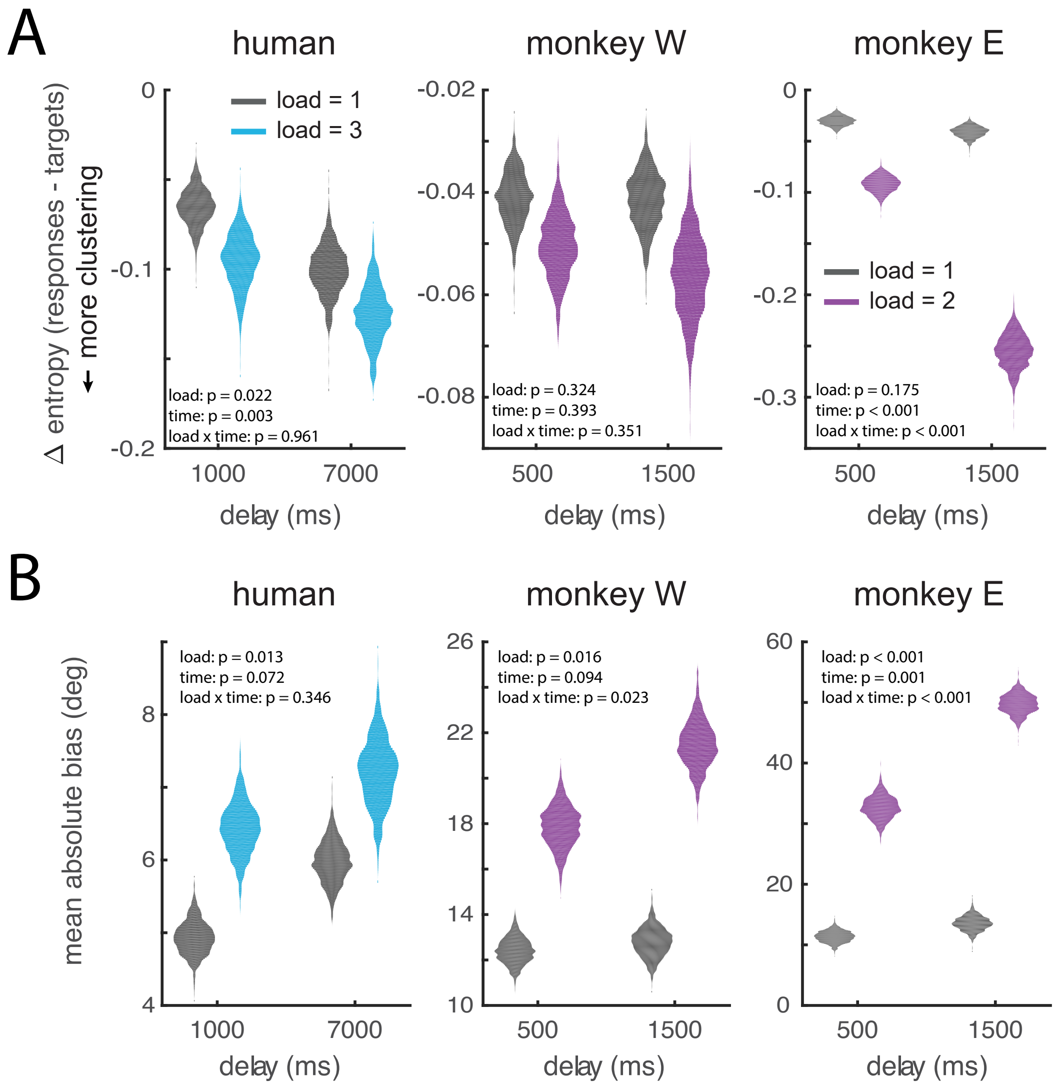

Supplementary Figure 1: Clustering increases with load and delay. (A) Difference in entropy between the response distribution and target distribution for humans and monkeys as a function of load and delay. More negative values indicate more clustered memory reports. (B) Mean absolute bias (averaged across all target colors) for humans and monkeys as a function of load and delay. Violin plots indicate distribution of bootstrapped values. P-values reflect non-parametric regression (bootstrap). Source data are provided as a Source Data file.

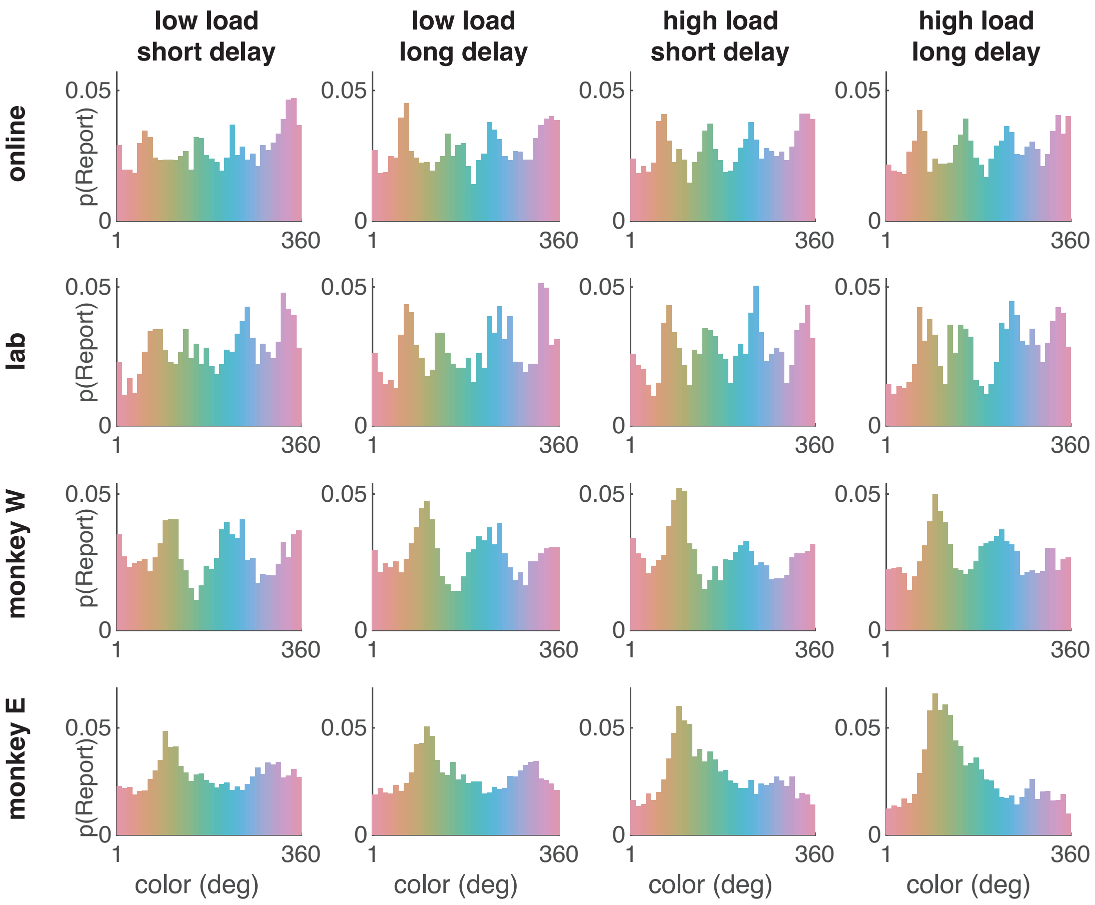

Supplementary Figure 2: Response histograms for humans and monkeys by condition. Source data are provided as a Source Data file.

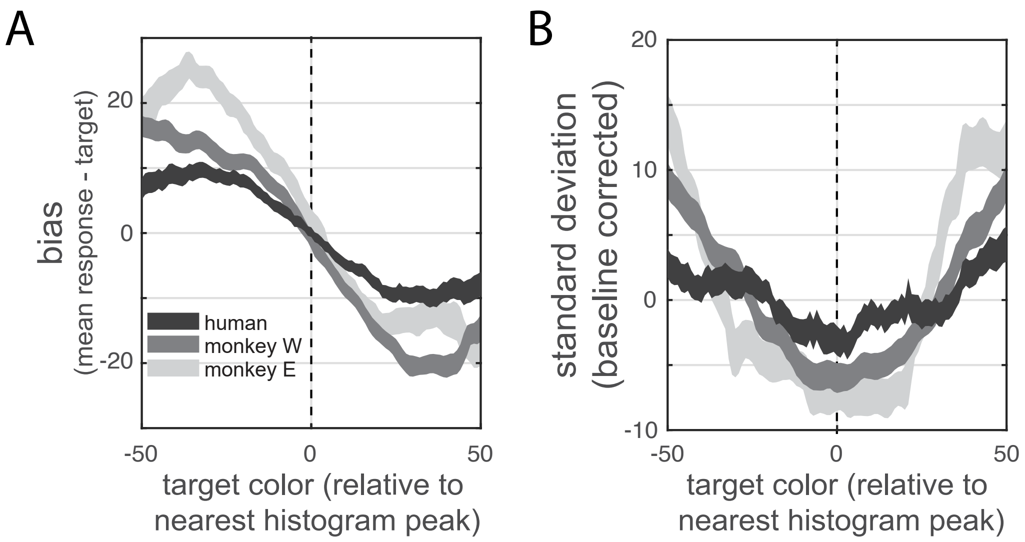

Supplementary Figure 3: Bias and standard deviation of memory reports around putative attractors. Putative attractors are identified as significant peaks in subjects' distribution of reported colors. Error bars reflect standard error of the mean. Source data are provided as a Source Data file.

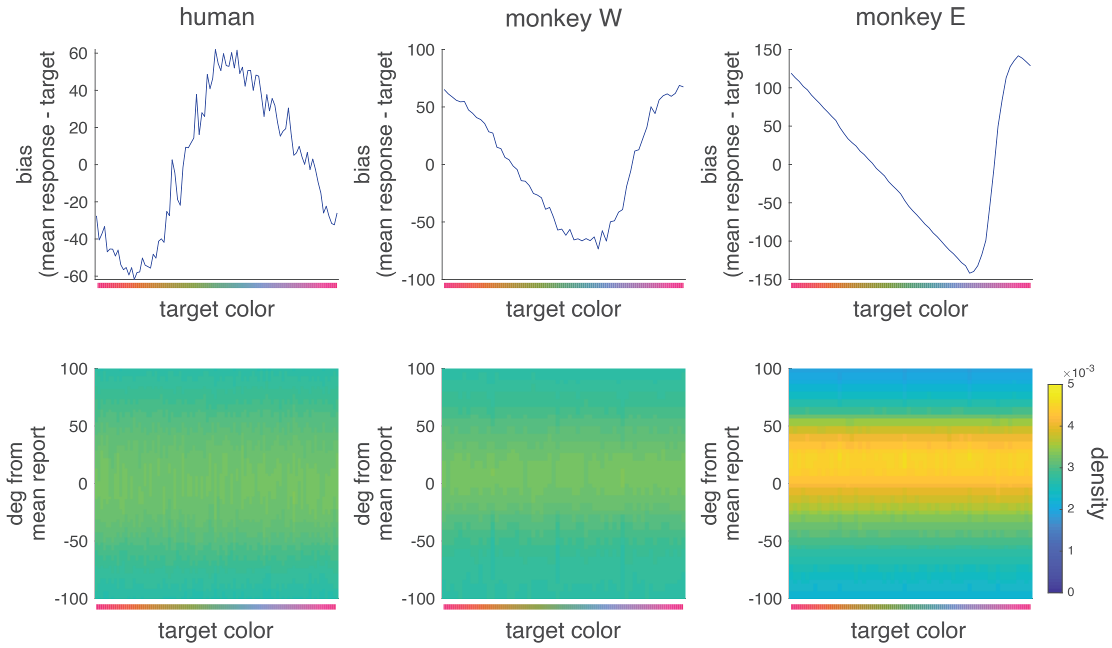

Supplementary Figure 4: Simulated performance of a non-uniform guessing strategy. In the non-uniform guessing strategy, the subject reports one of the frequently-reported colors on a subset of trials, and the color reported is independent from the identity of the target (see Methods). Plots show the expected pattern of bias (top row) and precision around mean report (bottom row) as a function of target color. For the subset of trials on which the subject makes a non-uniform guess, bias depends only on the distance between the target color and the mean reported color across all trials (top row). Additionally, precision does not vary as a function of target color (bottom row). Source data are provided as a Source Data file.

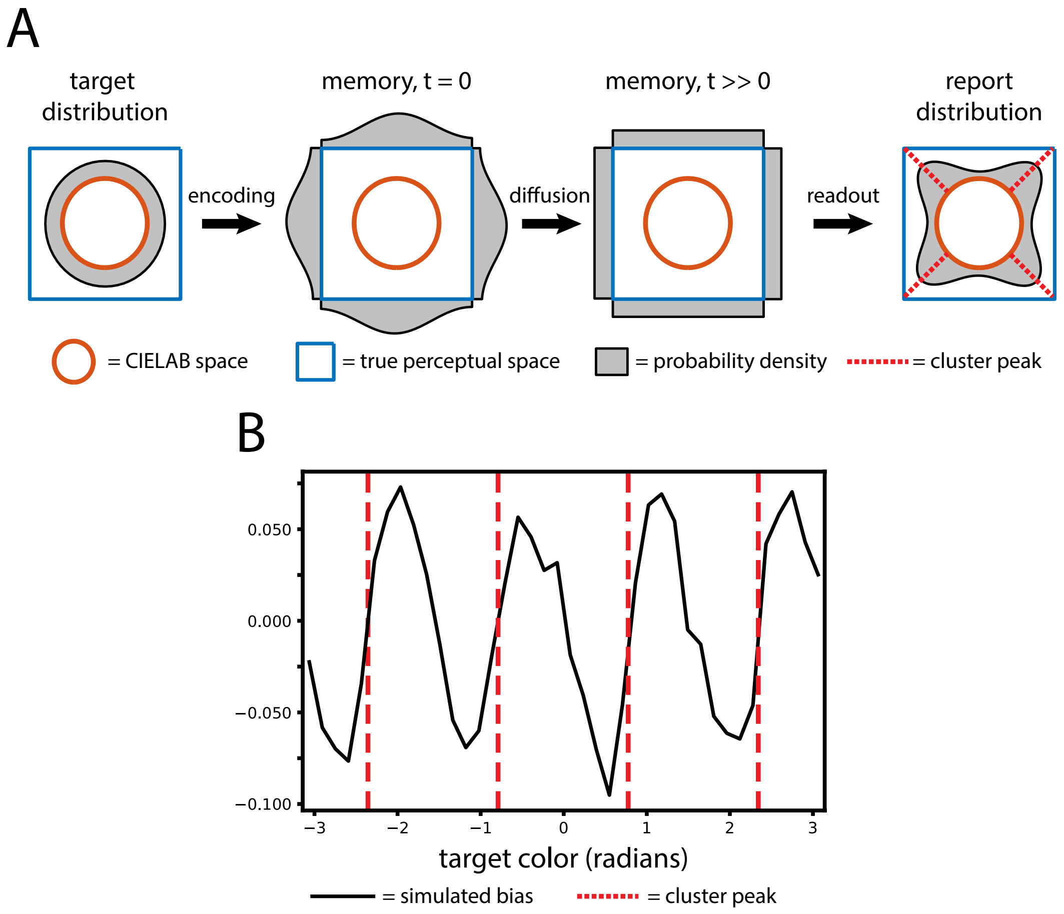

Supplementary Figure 5: Simulated performance based on a nonlinear encoding of CIELAB color space. A nonlinear mapping between stimulus space and a subject's true representational space introduces clustering into memory reports without discrete attractor dynamics but cannot explain memory biases. (A) Model structure. Far left: across trials, target colors are uniformly distributed in CIELAB space (orange circle). Center left: true perceptual space is assumed to be any arbitrary shape (here: a square) other than a circle concentric with CIELAB space. When the uniform target colors are projected into this true space, clusters form at locations where changes in the CIELAB angle result in small changes in the true space. Center right: random diffusion in memory erodes the concentration gradient in the true perceptual space over time. For clarity, we show a complete erosion of the concentration gradient at $t >> 0$, but in practice the concentration gradient will only partially degrade for delays of a few seconds when reports are still reasonably accurate. Far right: Projecting the uniform distribution of memories in true space back into CIELAB space results in clustering at locations where changes in the true space result in small changes in θ. For a square perceptual space, this results in clustering at vertex angles, which may be mistaken for attractors. (B) Predicted bias based on 100,000 simulated trials. Counterintuitively, this model predicts repulsive (positive slope) bias around points of peak clustering, inconsistent with empirical results (Fig. 3). We thank an anonymous reviewer for proposing and implementing this alternative model. Source data are provided as a Source Data file.

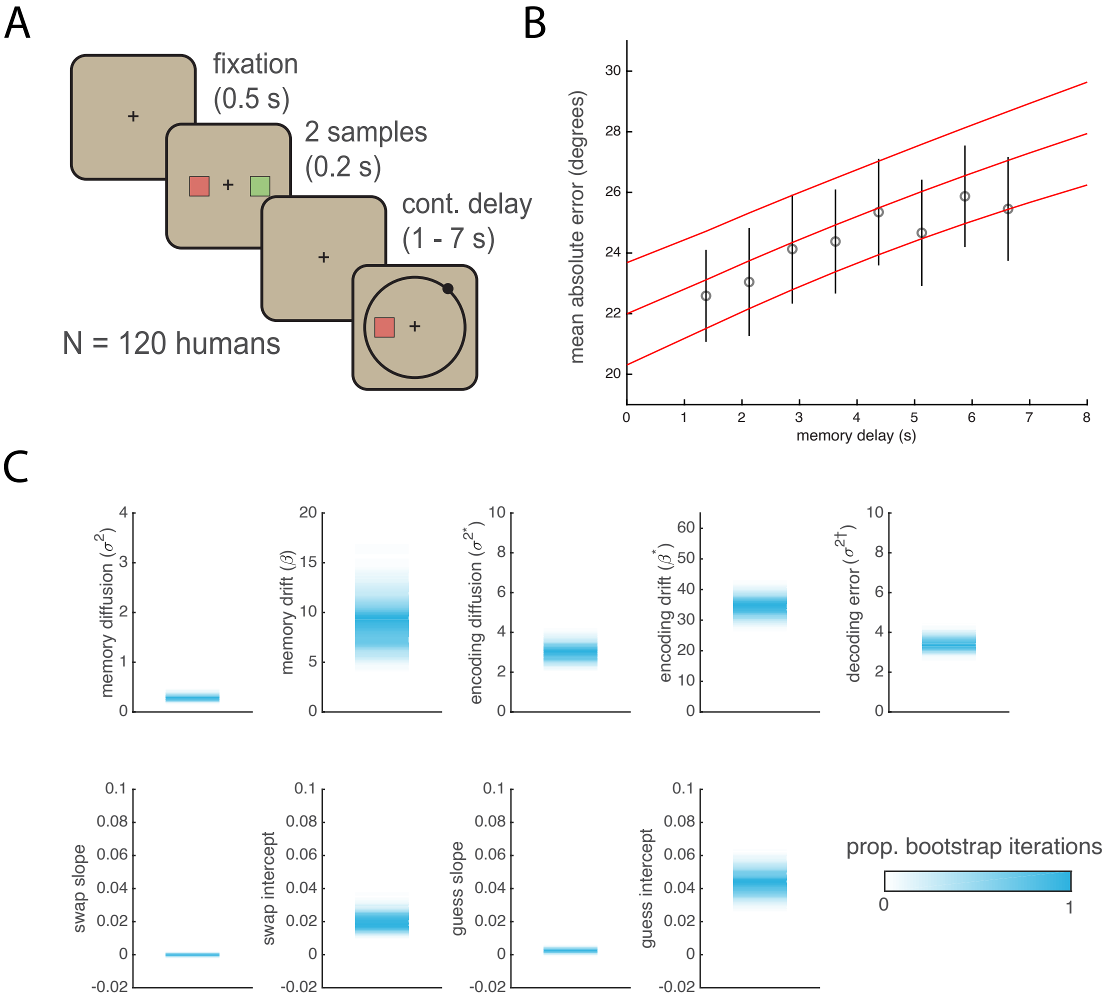

Supplementary Figure 6: Experiment 1b design and results. (A) Experiment 1b design. Experiment 1b was similar to 1a, except that there were always two samples and the delay varied continuously between 1 and 7 seconds (see methods). (D) Mean absolute error +/- 95% CI (bootstrap) as a function of delay length. Red = model fit, black = data. (C) Maximum likelihood dynamic model parameter estimates for Experiment 1b. Color intensity reflects normalized proportion of bootstrap iterations. Source data are provided as a Source Data file.

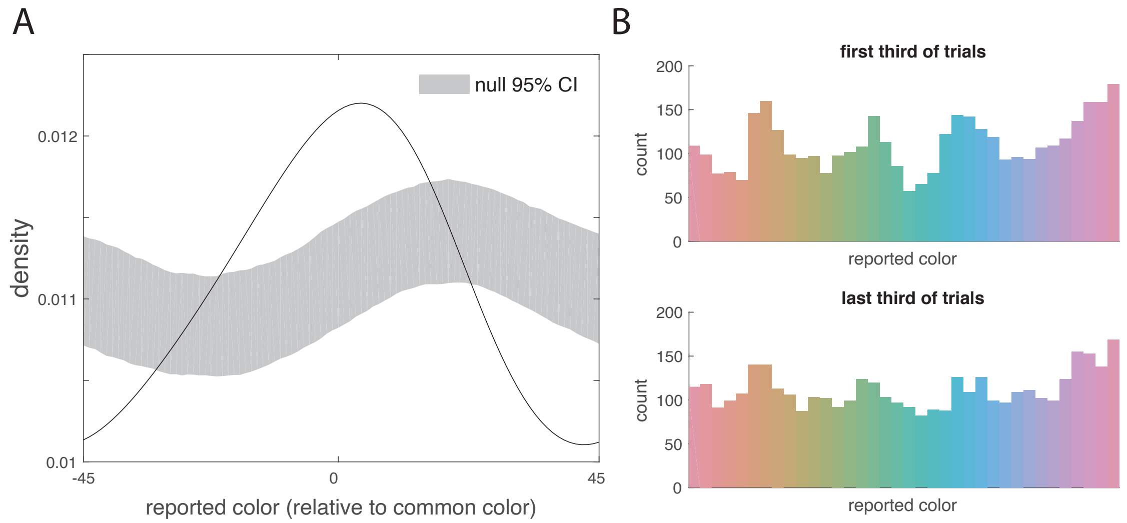

Supplementary Figure 7: Distribution of color reports in Experiment 2. (A) Probability of report relative to common color location in colorspace, computed using the subset of trials in which target colors were distributed uniformly. (B) Distribution of reported colors for the first and last third of trials, computed using the subset of trials in which target colors were distributed uniformly. Source data are provided as a Source Data file.

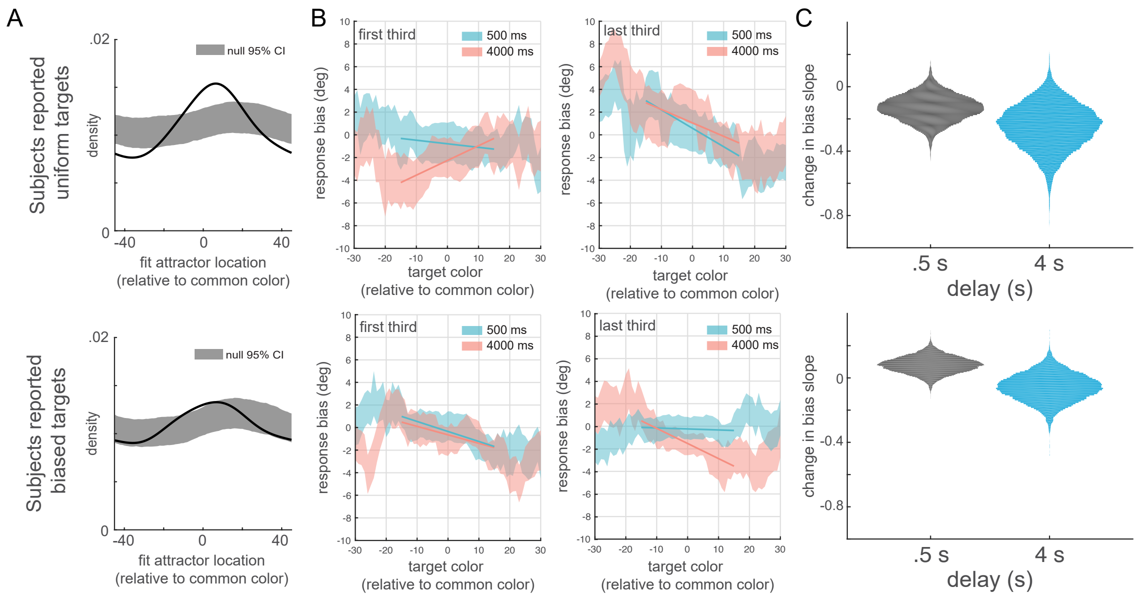

Supplementary Figure 8: Performance of Experiment 2 subjects, grouped by debriefing report. Regardless of whether experiment 2 subjects incorrectly reported that the distribution of target colors was unbiased (top row) or correctly reported that the distribution of target colors was biased (bottom row), both groups were more likely than chance to display attractors at common color locations (A) and both groups showed a numerical trend for slope to decrease more on long-delay trials (B-C). Source data are provided as a Source Data file.

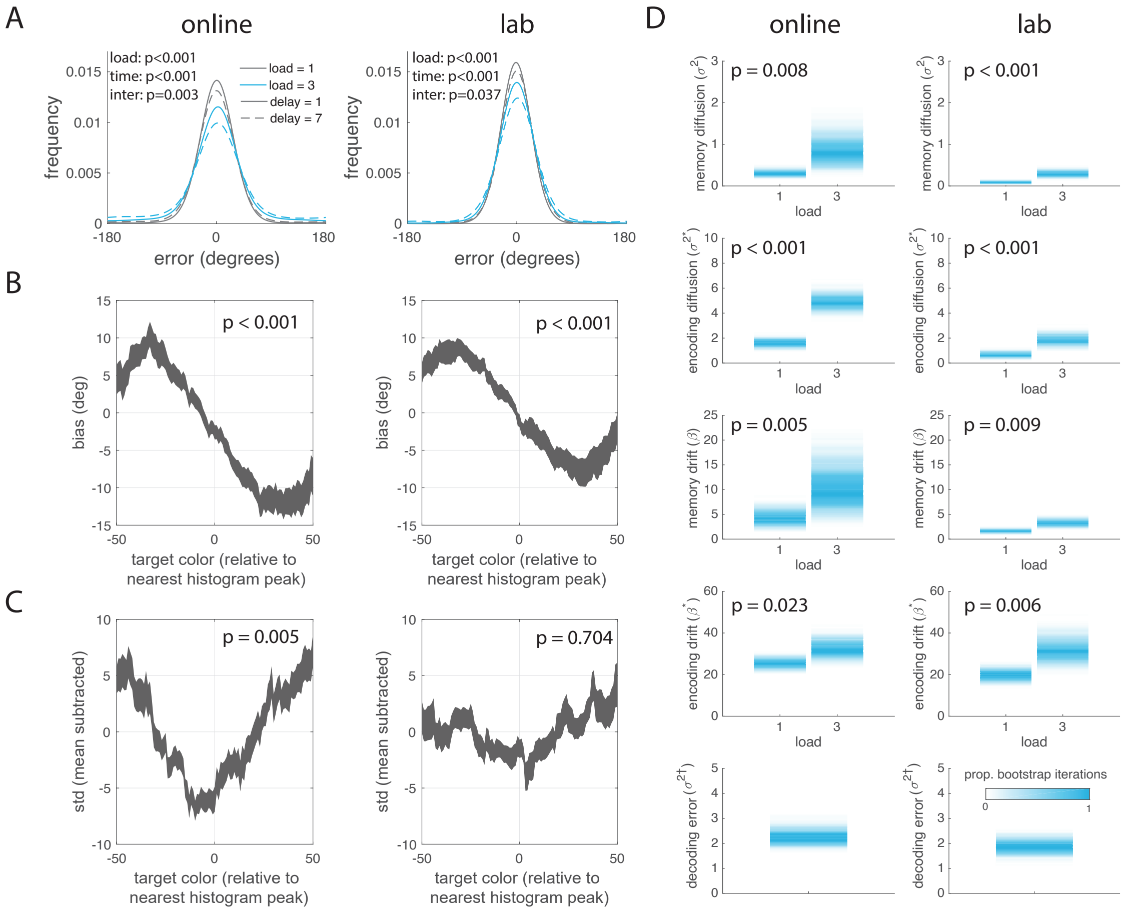

Supplementary Figure 9: Online and lab subjects show qualitatively similar behavior. (A) Distribution of angular error. P-values reflect the results of a repeated-measures ANOVA predicting mean error as a function of load and time, as in text describing Fig. 1b. (B) Bias around putative attractors. P-values reflect a t-test of the slope of bias at histogram peaks vs zero, as in text describing Fig. S3. (C) Precision around putative attractors. P-values reflect a t-test of the relative standard deviation of memory reports at histogram peaks vs zero, as in text describing Fig. ED5. (D) Dynamical model parameter fits. P-values reflect differences in diffusion and drift parameters as a function of load, as in the text describing Fig. 6. Source data are provided as a Source Data file.

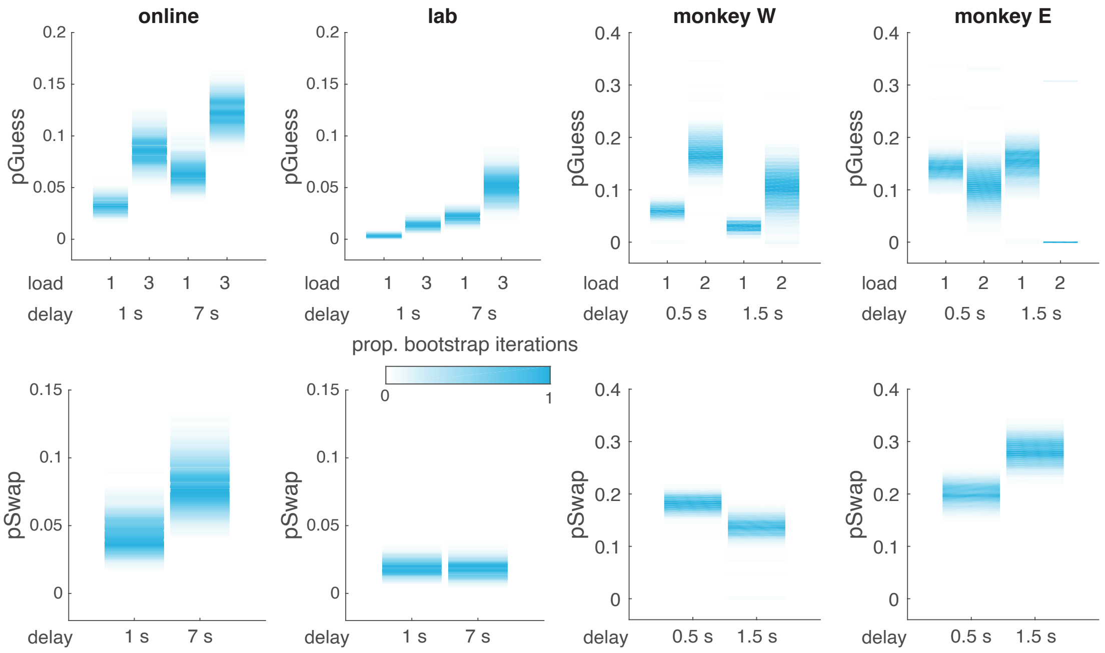

Supplementary Figure 10: Estimated rate of guessing and swap errors. Plots show the maximum likelihood guess and swap probabilities from dynamic model fits for each load and delay. Color intensity reflects normalized proportion of bootstrap iterations. Source data are provided as a Source Data file.

population	model	no. param	AIC	ΔAIC	wAIC	BIC	ΔBIC	wBIC
human	full	27	119885	0	1.00	120094	0	0.98
	drop β_L	25	119908	23	0.00	120102	7	0.02
	drop β_L^*	25	120500	615	0.00	120694	600	0.00
online	full	27	85108	0	0.99	85308	6.2	0.04
	drop β_L	25	85117	8.6	0.01	85302	0	0.96
	drop β_L^*	25	85502	393	0.00	85686	385	0.00
lab	full	27	34016	0	1.00	34194	0	0.99
	drop β_L	25	34038	22	0.00	34203	8.8	0.01
	drop β_L^*	25	34310	294	0.00	34475	281	0.00
monkey W	full	26	129087	0	1.00	129286	0.0	0.80
	drop β_L	24	129105	18	0.00	129289	2.8	0.20
	drop β_L^*	24	129654	567	0.00	129838	552	0.00
monkey E	full	26	144746	0	1.00	144947	0	1.00
	drop β_L	24	144873	126	0.00	145058	111	0.00
	drop β_L^*	24	144871	125	0.00	145057	110	0.00

Supplementary Table 1: AIC and BIC model comparison. We compared the full model with competing models without attractor dynamics during encoding or maintenance. Model weights (wAIC and wBIC) indicate the probability that the given model is the best model in the set given the data and set of candidate models. Source data are provided as a Source Data file.

subject	full	drop β_L	drop β_L^*
human	15.3	14.7	0
monkey W	14.5	14.1	0.6
monkey E	5.0	2.0	1.9

Supplementary Table 2: Cross-validated model comparison. Mean difference in 20-fold cross-validated log-likelihood for full model and competing models without attractor dynamics during encoding or maintenance. Values represent the increase in log-likelihood relative to the worst fitting model, averaged across folds. Source data are provided as a Source Data file.

subject	model	p(Guess) eq.				parameter MLE							
		C	L	D	I	σ_1	σ_2	σ_1^*	σ_2^*	β_1	β_2	β_1^*	β_2^*
monkey W	1	x	x	x	x	15	31	12	13	4	15	33	45
	2	x	x	x		15	29	12	14	4	14	32	44
	3	x		x		15	36	11	17	5	17	32	49
	4	x	x			13	26	14	16	4	13	32	44
	5	x				12	32	14	21	4	16	31	50
	6					13	21	32	69	0	9	161	344
monkey E	1	x	x	x	x	17	39	48	58	28	45	8	82
	2	x	x	x		23	35	44	71	33	39	4	88
	3	x		x		23	35	47	57	32	44	2	85
	4	x	x			17	29	47	76	29	34	6	84
	5	x				17	30	52	60	28	40	4	80
	6					17	29	69	76	25	35	5	85

Supplementary Table 3: Parameter fits for simplified models of guessing and swap behavior. Maximum likelihood estimates for drift and diffusion parameters for models with different parameterizations of guessing probability. An ‘x’ indicates that a parameter is included in a given model. For the most flexible model (model 1, identical to that reported in the main text), guessing is effectively parameterized by a constant term C, a coefficient determining an effect of load on guessing (L), a coefficient determining an effect of memory delay on guessing (D), and an interaction term (I). Successive models drop combinations of these terms, yielding less flexibility in how guessing changes with load and time. For example, for model 5, p(Guess) is constant across load and time. Regardless of the parameterization, however, drift and diffusion consistently increase with load during both encoding and memory.
