# Supplementary material for: Error-correcting dynamics in visual working memory
Source: Nat Commun. 2019 Jul 29;10:3366. doi: 10.1038/s41467-019-11298-3 (PMC6662698; doi:10.1038/s41467-019-11298-3)
Supplement: Supplementary file 3 — Reporting Summary [file 41467_2019_11298_MOESM3_ESM.pdf]

nature research

Corresponding author(s): *Double-blind peer review submissions: write DBPR and your manuscript number here instead of author names.*

Last updated by author(s): *YYYY-MM-DD*

# Reporting Summary

Nature Research wishes to improve the reproducibility of the work that we publish. This form provides structure for consistency and transparency in reporting. For further information on Nature Research policies, see Authors & Referees and the Editorial Policy Checklist.

## Statistics

For all statistical analyses, confirm that the following items are present in the figure legend, table legend, main text, or Methods section.

| n/a | Confirmed | |
|---|---|---|
| ☐ | ☒ | The exact sample size ($n$) for each experimental group/condition, given as a discrete number and unit of measurement |
| ☐ | ☒ | A statement on whether measurements were taken from distinct samples or whether the same sample was measured repeatedly |
| ☐ | ☒ | The statistical test(s) used AND whether they are one- or two-sided<br>*Only common tests should be described solely by name; describe more complex techniques in the Methods section.* |
| ☒ | ☐ | A description of all covariates tested |
| ☐ | ☒ | A description of any assumptions or corrections, such as tests of normality and adjustment for multiple comparisons |
| ☐ | ☒ | A full description of the statistical parameters including central tendency (e.g. means) or other basic estimates (e.g. regression coefficient) AND variation (e.g. standard deviation) or associated estimates of uncertainty (e.g. confidence intervals) |
| ☐ | ☒ | For null hypothesis testing, the test statistic (e.g. $F$, $t$, $r$) with confidence intervals, effect sizes, degrees of freedom and $P$ value noted<br>*Give P values as exact values whenever suitable.* |
| ☒ | ☐ | For Bayesian analysis, information on the choice of priors and Markov chain Monte Carlo settings |
| ☐ | ☒ | For hierarchical and complex designs, identification of the appropriate level for tests and full reporting of outcomes |
| ☐ | ☒ | Estimates of effect sizes (e.g. Cohen's $d$, Pearson's $r$), indicating how they were calculated |

*Our web collection on statistics for biologists contains articles on many of the points above.*

## Software and code

Policy information about availability of computer code

| | |
|---|---|
| Data collection | Laboratory experiments were programmed in MATLAB using Psychtoolbox. Online experiments were programmed in javascript and run using psiTurk. |
| Data analysis | Data were analyzed using custom scripts in MATLAB. |

For manuscripts utilizing custom algorithms or software that are central to the research but not yet described in published literature, software must be made available to editors/reviewers. We strongly encourage code deposition in a community repository (e.g. GitHub). See the Nature Research guidelines for submitting code & software for further information.

## Data

Policy information about availability of data

All manuscripts must include a data availability statement. This statement should provide the following information, where applicable:

- Accession codes, unique identifiers, or web links for publicly available datasets
- A list of figures that have associated raw data
- A description of any restrictions on data availability

All data that support the findings of this study are available from the corresponding author upon request.

# Field-specific reporting

Please select the one below that is the best fit for your research. If you are not sure, read the appropriate sections before making your selection.

☐ Life sciences ☒ Behavioural & social sciences ☐ Ecological, evolutionary & environmental sciences

# Behavioural & social sciences study design

All studies must disclose on these points even when the disclosure is negative.

| | |
|---|---|
| Study description | Quantitative psychophysical study of working memory. Subjects included both humans and non-human primates. |
| Research sample | The sample consisted of (1) Princeton University undergraduates and (2) adults from throughout the United States who participated online via Amazon Mechanical Turk. 35 women and 38 men (mean age 31.7 years) participated in experiment 1 online. 17 female and 16 male Princeton undergraduates (mean age 20.3 years) participated in experiment 1. 73 women and 82 men (mean age 33.3 years) participated in experiment 2 online. These samples are representative of their respective populations. Study sample was chosen based on availability. |
| Sampling strategy | N = 2 is the standard in the field for non-human primates. For humans, the sample size was chosen based on preliminary analysis of an independent pilot study with N=57 subjects. |
| Data collection | Laboratory participants completed the task on a computer while eye position was monitored continuously. Experiments were carried out in a dedicated testing suite while a researcher monitored the signal quality of a video-based eyetracker. The researcher was not blind to the study hypothesis during data collection. Online participants completed the task on a computer. |
| Timing | Data were collected from February 2017 to January 2018. |
| Data exclusions | Details in Methods. Briefly, human subjects who were estimated to be randomly guessing on more than 20% of trials were excluded from analysis. This threshold of 20% was set independently based on analysis of a separate pilot cohort. No monkey data were excluded from analysis. |
| Non-participation | No participants declined participation. |
| Randomization | Subjects were not assigned to groups in Experiment 1. Subjects were randomly assigned 4 'common colors' in Experiment 2 (see Methods) . |

# Reporting for specific materials, systems and methods

We require information from authors about some types of materials, experimental systems and methods used in many studies. Here, indicate whether each material, system or method listed is relevant to your study. If you are not sure if a list item applies to your research, read the appropriate section before selecting a response.

## Materials & experimental systems

| n/a | Involved in the study | |
|---|---|---|
| ☒ | ☐ | Antibodies |
| ☒ | ☐ | Eukaryotic cell lines |
| ☒ | ☐ | Palaeontology |
| ☐ | ☒ | Animals and other organisms |
| ☐ | ☒ | Human research participants |
| ☒ | ☐ | Clinical data |

## Methods

| n/a | Involved in the study | |
|---|---|---|
| ☒ | ☐ | ChIP-seq |
| ☒ | ☐ | Flow cytometry |
| ☒ | ☐ | MRI-based neuroimaging |

## Animals and other organisms

Policy information about studies involving animals; ARRIVE guidelines recommended for reporting animal research

| | |
|---|---|
| Laboratory animals | Monkey subjects were two 9-year old male rhesus macaques. |
| Wild animals | N/A |
| Field-collected samples | N/A |
| Ethics oversight | All animal procedures were done with approval from and in accordance with the policies and procedures of the Princeton University Institutional Animal Care and Use Committee. |

Note that full information on the approval of the study protocol must also be provided in the manuscript.

## Human research participants

Policy information about studies involving human research participants

| | |
|---|---|
| Population characteristics | As described above, our population consisted of (1) Princeton University undergraduates and (2) adults from throughout the United States who participated online via Amazon Mechanical Turk. 35 women and 38 men (mean age 31.7 years) participated in experiment 1 online. 17 female and 16 male Princeton undergraduates (mean age 20.3 years) participated in experiment 1. 73 women and 82 men (mean age 33.3 years) participated in experiment 2 online. These samples are representative of their respective populations. Study sample was chosen based on availability. |
| Recruitment | Participants were recruited using advertisements on the Princeton University psychology subject pool website, and advertisements on the Amazon Mechanical Turk website. |
| Ethics oversight | Protocol was approved by the Princeton University Institutional Review Board. |

Note that full information on the approval of the study protocol must also be provided in the manuscript.
